# Mechanism of Accelerated Deterioration of High-Temperature Vulcanized Silicone Rubber under Multi-Factor Aging Tests Considering Temperature Cycling

**DOI:** 10.3390/polym15153210

**Published:** 2023-07-28

**Authors:** Shiyin Zeng, Wendong Li, Yanan Peng, Yucheng Zhang, Guanjun Zhang

**Affiliations:** State Key Laboratory of Electrical Insulation and Power Equipment, Xi’an Jiaotong University, Xi’an 710049, China

**Keywords:** silicone rubber, temperature cycling, crosslink, degradation, corona discharge, moisture

## Abstract

High-temperature vulcanized silicone rubber (HTV-SR) employed for composite insulators is continuously subjected to a complex environment of alternating heat, corona discharge, humidity, etc. These stresses (especially alternating heat) complicate the aging mechanism of HTV-SR, which lacks systematic investigation. In this paper, a multi-factor aging platform considering temperature cycling, moisture, and corona discharge is established. Specifically, four temperature-cycling settings are employed, each of which lasts for 15 cycles. The surface morphology, hydrophobicity, and chemical, mechanical, and electrical properties of aged samples are methodically characterized. Experimental results show that the aging degree is correlated to the range of temperature cycling, which is attributed to diverse crosslink-degradation degrees with different temperature differences. Under a large temperature difference (70 °C), HTV-SR possesses a high crosslinking degree and a low degradation degree, making the material hard but easy to crack with alternating thermal stress. Then, severe defects and water condensation emerge on the HTV-SR surface, which promote the diffusion of corona products and water molecules into the material. The subsequent rise in crosslinking density caused by in-depth oxidation further exacerbates the aging of the material. Consequently, it brings about poor hydrophobicity, high interfacial polarization, and shallow trap energy levels in HTV-SR. This work provides a detailed analysis of the aging mechanism of HTV-SR in a simulated on-site environment.

## 1. Introduction

High-temperature vulcanized silicone rubber (HTV-SR) is applied as the sheath material of composite insulators for its superior insulation performance and high resistance to pollution flashover [1,2]. Nevertheless, the mechanical and electrical performance of HTV-SR deteriorates after long-term exposure to various environmental stresses [3,4]. The resulting aging problems are one of the main causes of composite insulator failures, endangering the security of grid operation. Thus, it is necessary to carry out more simulated field research on HTV-SR for a proper explanation of multi-factor aging and associated potential failures [5].

To date, great efforts have thus been undertaken in artificially accelerated aging studies of HTV-SR. Some effective methods, such as the 5000 h multi-factor aging test [6], inclined plane test, and tracking wheel test [7], were proposed to simulate the harsh environment of HTV-SR aging. However, these pioneering efforts have focused mainly on testing procedures without much attention to mechanistic interpretation. Based on these aging techniques, research in the last decade has started to focus on the aging mechanism of HTV-SR, i.e., how different aging stresses affect the material properties. Hence, a variety of single-factor aging analyses involving heat [8,9], ultraviolet (UV) radiation [10,11], corona [12,13], and tensile stress were carried out, and important conclusions were drawn [14]. Nevertheless, the multi-factor aging of HTV-SR has always been a complex issue, and the setting of its aging conditions determines whether it can properly simulate the aging in the natural environment. To the best of our knowledge, only a few researchers have taken on the challenge of building innovative multi-factor aging platforms for HTV-SR. Bi et al. [15] analyzed the corona aging characteristics of SR under different humidity, salt fog, and haze conditions, demonstrating the facilitating role that humidity plays in the deterioration of SR. Hu et al. [16] studied the aging mechanism of SR under both thermal and mechanical stresses, confirming the competitive and synergistic effects between the two stresses. Xu et al. [17] established an aging platform combining an electric field and hydrothermal conditions for HTV-SR and discussed the failure law based on the trap theory. According to the studies above, high voltage, heat, and moisture are the most common factors for HTV-SR aging, whereby the setting of temperature is usually constant [18,19].

There have been attempts to utilize alternating temperatures in the artificially accelerated aging of HTV-SR, since it can better mimic the periodical fluctuation of on-site temperature. Cui et al. [20] noted significant thermal expansion and contraction effects of liquid SR seals under temperature-cycling conditions. Due to similar material structure, defects resulting from possible thermal deformation of HTV-SR at such alternating temperatures are fatal to the bond between the sheath/skirt and the core rod of composite insulators [21]. Moreover, some industries, such as Mitsubishi Electric, believe that a cycling condensation test is needed to assess the reliability of products [22]. The water condensation phenomenon, which exacerbates the aging effects of moisture, is more likely to form under temperature-cycling conditions with high humidity [23]. To sum up, the influence of temperature cycling (e.g., temperature difference (ΔT) and average temperature) on the degradation behaviors of HTV-SR is significant but has not been thoroughly discussed. Furthermore, research on the multi-factor aging of HTV-SR, especially considering cycling thermal stress, is still lacking.

Therefore, in this paper, a multi-factor aging test method considering temperature cycling, corona discharge, and high humidity is developed to simulate the on-site aging of HTV-SR in composite insulators. Then, surface morphology, hydrophobicity, chemical structures, and mechanical and electrical properties of aged specimens are characterized. Among them, the equilibrium swelling test and Fourier transform infrared (FTIR) spectra are employed to quantify the degree of crosslink and degradation, respectively. Based on the experimental results, the effects of temperature cycling on the aging mechanism and aging behaviors of HTV-SR are summarized.

## 2. Experimental Details

### 2.1. Sample Preparation

The HTV-SR samples were manufactured by Xiangyang Guowang Composite Insulators Co., Ltd., Xiangyang, China. The formulation of the HTV-SR sample was 100 parts pure methyl vinyl silicone rubber, 33 parts 8 μm sized fumed silica (specific surface area: 195 m^2^/g), 130 parts 1.7 μm sized aluminum hydroxide powder, and 6 parts hydroxy silicone oil in weight. These raw materials were placed into the kneader for mixing at a speed frequency of 50 Hz and heat treatment at 150 °C. The homogenized rubber was then filtered and cooled to room temperature. Afterwards, about 1 part of 2,5-dimethyl-2,5-di (t-butyl peroxy) hexane in weight was mixed into the rubber and heated (less than 70 °C) for vulcanization. As a result, HTV-SR samples of 60 × 60 × 1 mm^3^ size were prepared.

### 2.2. Multi-Factor Aging of HTV-SR

**Figure 1:**
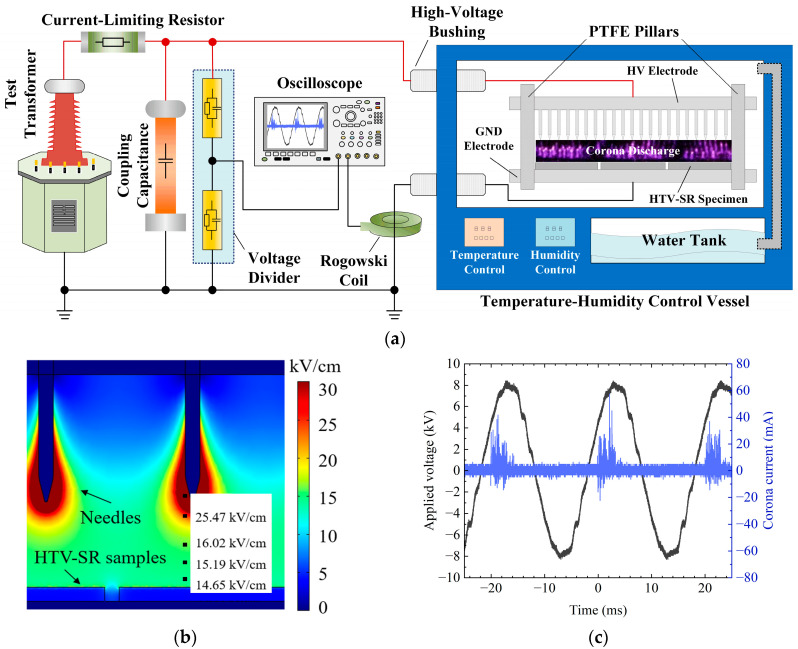
Multi-factor accelerated aging platform: (**a**) temperature-humidity control vessel; (**b**) the electric field simulation image of a multi-needle plate electrode; (**c**) voltage-current waveforms of corona discharge.

A multi-factor accelerated aging platform considering temperature cycling, relative humidity, and corona discharge is illustrated in Figure 1a. The multi-needle plate electrode system has about 441 stainless-steel needles to produce a sufficient area for corona discharge. The length of one stainless-steel needle is 9 mm, and its horizonal distance to an adjacent needle is also 9 mm. HTV-SR sheets are placed on the downward stainless-steel plate. The vertical gap between the needle tip and the sample surface is 7 mm.

During corona aging, the applied voltage was 8.44 kV. Then, great strength in corona discharge could be obtained without breakdown of the air gap between the needle tip and the sample surface. To observe the electric field distribution at the needle tip, a simulation model was built using COMSOL. The discharge image and simulation image of a multi-needle plate electrode are shown in Figure 1b. In addition, the applied voltage and corona current were measured, and the results are shown in Figure 1c. Then, the corona power of 0.2 W was determined by integrating 100 corona discharge pulses [15].

The temperature and relative humidity of the aging environment were controlled in the temperature-humidity control vessel. It should be noted that the vessel can only control the relative humidity to be constant in the temperature range of 15~85 °C, and the corresponding humidity is controlled in the range of 20~95%. Due to technical limitations, it is the most severe temperature condition that the equipment can achieve at present. Therefore, four groups of temperature-cycling tests were set up, which are shown in Table 1. Each group contains 3 samples of HTV-SR. Among them, three groups denoted with “CH” controlled the corona (C) power to be 0.2 W and the relative humidity (H) to be 50%, respectively. As a control, the group denoted with “N” was placed in an oven without controlling the corona discharge or the relative humidity. For each group, the thermal cycle frequency was approximately 16 h per cycle (7 h for high temperature, 7 h for low temperature, 0.5 h for each rise or fall of temperature, and 2 h for the total rise or fall of temperature), and the total aging time was 15 cycles.

### 2.3. Characterization

#### 2.3.1. Surface Morphology and Hydrophobicity

Visual changes on the surface were observed with an optical microscope (OLYMPUS SZ61, Olympus, Tokyo, Japan) microscopic surface changes of the sample were examined by scanning electron microscopy (SEM, VE-9800S, Keyence, Osaka, Japan). The magnification was 500×, and the applied voltage was 2 kV.

The contact angle measuring instrument (JY-82, Dedu Instrument, Changzhou, China) was applied to perform hydrophobicity tests, and the static contact angle of each specimen was measured 5 times to obtain an average.

#### 2.3.2. Chemical Properties

FTIR spectra (Nicolet IN10 + IZ10, Thermo Fisher Scientific, Waltham, MA, USA) were applied to identify chemical bonds in original and aged HTV-SR [24]. The test was measured 5 times to obtain an average. X-ray photoelectron spectra were evaluated through a photoelectron spectrometer (Thermo Scientific K-Alpha).

Crosslinking density can be used to quantitatively characterize the degree of crosslinking in HTV-SR. The data from each sample were measured twice to obtain an average. Based on the equilibrium swelling method [25], the swelling procedure was carried out by soaking tiny pieces of samples in methylbenzene for 7 days until fully swollen. First, the volume percentage of the specimen (*v*_2_) was calculated by the following equation:

(1) $$V_{2}=\frac{m_{1}b/\rho_{1}}{m_{1}b/\rho_{1}+(m_{2}-m_{1})\rho_{s}}$$

where *m*_1_ is the initial quality of the specimen and *m*_2_ is the final quality after fully swelling the specimen; *b* is the mass percentage of siloxane in HTV-SR, which can be provided by the manufacturer; *ρ*_1_ and *ρ_s_* are the densities of the specimen and methylbenzene (*ρ_s_* = 0.866 g/cm^3^), respectively. Then, the crosslinking density of the HTV-SR specimen is

(2) $$D=\frac{-[\ln(1-v_{2})+v_{2}+\alpha v_{2}{}^{2}]}{2\rho_{s}V_{s}(v_{2}{}^{1/3}-v_{2}/2)}$$

where *α* is the interaction parameter between HTV-SR and methylbenzene (*α* = 0.545), and *V_s_* is the molar volume of methylbenzene (*V_s_* = 106.7 cm^3^/mol).

#### 2.3.3. Mechanical Properties

To evaluate the mechanical properties of HTV-SR, hardness and elongation at break were tested. The hardness of specimens was measured by a Shore durometer (LX-A, Yuanfeng Testing, Yangzhou, China). Tensile properties were explored using the microcomputer-controlled electron universal testing machine (5 kN), and a dumbbell-shaped IV mold was adopted based on GB/T 528-2009. The elongation at break and hardness of each specimen were measured 5 times to obtain an average.

#### 2.3.4. Electrical Properties

To analyze the electrical properties of samples aged at different conditions, the dielectric constant, the dielectric loss, and the trap properties were considered. The dielectric spectra were characterized with a broadband dielectric spectrometer (Novocontrol Concept 80, Novocontrol, Montabaur, Germany) ranging from 10^0^ Hz to 10^6^ Hz. On the basis of the device designed by our group, the trap properties were obtained by the isothermal surface potential decay (ISPD) method [26].

## 3. Results

### 3.1. Surface Morphology and Hydrophobicity

#### 3.1.1. Optical Micrograph

**Figure 2:**
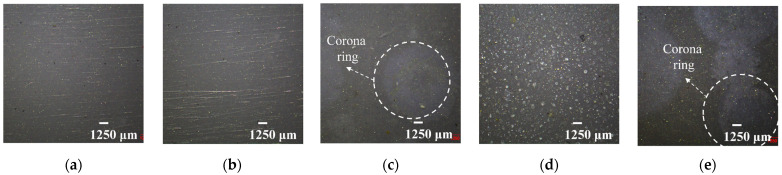
Microscopy photos of HTV-SR samples under different aging conditions: (**a**) virgin HTV-SR; (**b**) N-35~85 °C; (**c**) CH-15~65 °C; (**d**) CH-15~85 °C; (**e**) CH-35~85 °C.

Figure 2 presents the microscopy photos of the material surface before and after aging, and some visible changes are observed. Compared to virgin HTV-SR, Figure 2b shows that samples that undergo only cycling thermal stress barely change. As can be seen in Figure 2c,e, with the addition of corona discharge and humidity, white corona rings are uniformly distributed on the surface of samples. Unexpectedly, Figure 2d shows that with a large ΔT, corona rings vanish but chalking patches clearly emerge. It is inferred that the phenomenon is caused by the range of temperature cycling. The results are further confirmed by the SEM tests below.

#### 3.1.2. SEM Test

**Figure 3:**
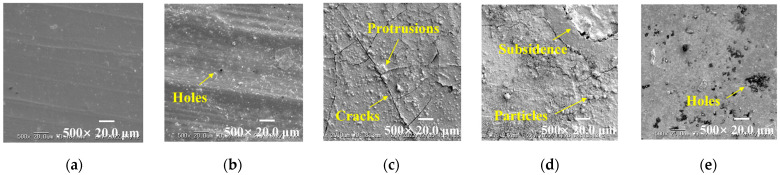
SEM photos of HTV-SR samples under different aging conditions: (**a**) virgin HTV SR; (**b**) N-35~85 °C; (**c**) CH-15~65 °C; (**d**) CH-15~85 °C; (**e**) CH-35~85 °C.

SEM photos of aged samples are shown in Figure 3. Different from the original flat surface, there are a few tiny holes on samples affected only by temperature. After adding the hydroelectric condition, surface defects clearly appear. As shown in Figure 3c, under the condition of the existence of a below-average temperature (15 °C), derived cracks and dense protrusions develop on the surface of samples. Despite the same ΔT, these surface defects are more serious than those of samples under only high temperatures in Figure 3e. Moreover, as ΔT increases to 70 °C, Figure 3d shows that large subsidence and obvious particles arise, contributing to the rough and flocculent surface. Combined with optical micrographs and SEM analysis, it can be judged that the severity of defects is positively correlated with ΔT, due to the thermal expansion or contraction effect of the HTV-SR matrix under temperature-cycling conditions.

#### 3.1.3. Hydrophobicity

**Figure 4:**
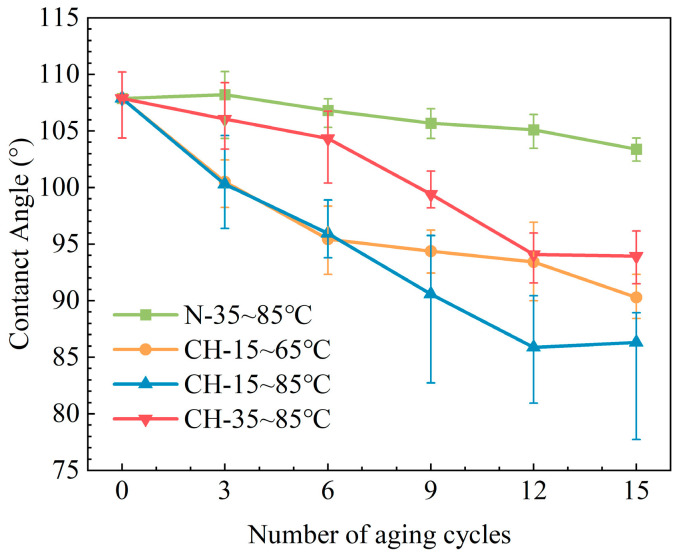
Variations in the hydrophobic water contact angle of HTV-SR.

As can be seen in Figure 4, under different conditions, the contact angle of HTV-SR samples gradually decreases with aging time. Under the influence of multiple stresses, such as corona discharge and humidity, the contact angles of samples decline rapidly. Among them, samples (CH-15~85 °C) with a larger ΔT are found to have poorer hydrophobicity. Based on the observation of surface morphology, it shows that once ΔT increases to 70 °C, permanent physical damages are developed. Then, a hydrophilic hydroxyl layer is likely to be built on the samples, significantly affecting the hydrophobicity of HTV-SR [10].

### 3.2. Chemical and Mechanical Properties

#### 3.2.1. Crosslinking Density from Equilibrium Swelling Test

**Figure 5:**
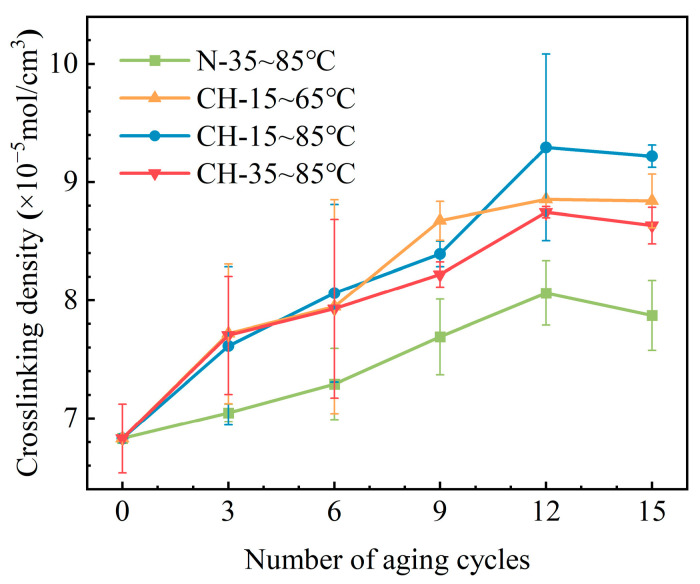
Crosslinking density changes of samples under different aging conditions.

In this section, the crosslinking density is used to characterize the crosslinking degree of samples, reflecting the number of crosslinking bonds [27]. Figure 5 shows the variation in crosslinking density of HTV-SR under different aging conditions. It is found that each variation curve for crosslinking density exhibits a trend of increasing and then slightly decreasing. In addition, there is a gradual slowdown in the growing rate of crosslinking density during the later stage of aging, which may be due to the increasing degree of molecular chain breakage. Compared to samples (N-35~85 °C) that undergo only temperature cycling, the crosslinking density of samples with the addition of hydroelectric conditions rises significantly. Additionally, when the number of aging cycles is greater than 9, samples of group CH-15~65 °C and group CH-15~85 °C obtain quite high crosslinking densities. The rise in crosslinking density is probably due to the in-depth oxidation as a result of corona and moisture exposure [28].

#### 3.2.2. Degree of Degradation from FTIR Test

**Figure 6:**
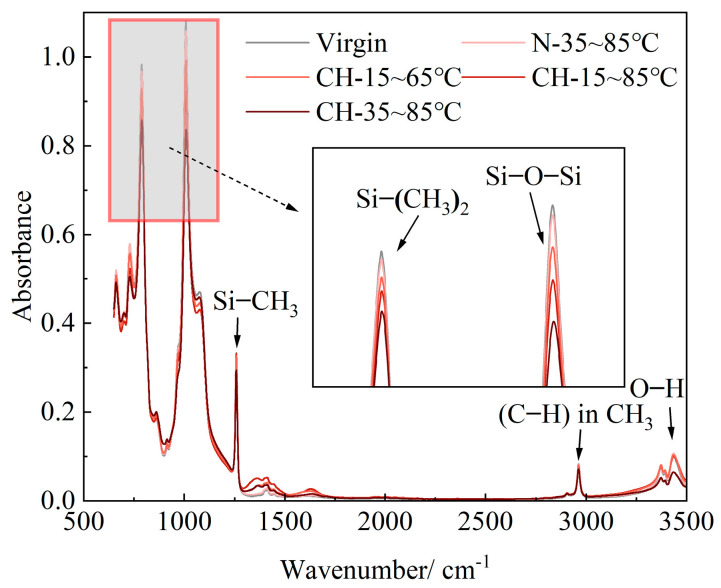
FTIR results of HTV-SR samples after 15 cycles of aging under different conditions.

Figure 6 presents the FTIR results of HTV-SR samples after 15 cycles of aging under different conditions. It shows that there are different degrees of reduction in the heights of the absorption peaks of Si−O−Si and Si−(CH_3_)_2_ on the main chain in the material.

**Figure 7:**
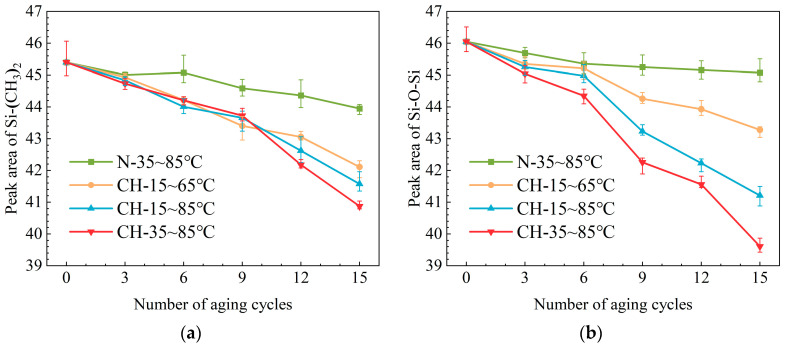
Peak area changes of chemical bonds in HTV-SR under different aging conditions: (**a**) Si−(CH_3_)_2_; (**b**) Si−O−Si.

In order to avoid the influence of background on the absorption peaks, the peak area method is adopted for the quantitative characterization of the corresponding groups [18]. Then, peak area changes of Si−(CH_3_)_2_ and Si−O−Si under different aging conditions are obtained by calculation. As shown in Figure 7, despite the similar trend of peak area changes, the descent speed of Si−(CH_3_)_2_ is slightly slower than that of Si−O−Si. In these two types of chemical bonds, the peak area of Si−(CH_3_)_2_ concentrates more on the structural changes of Si atoms. During the crosslinking reaction, highly oxygenated Si atoms, such as Si(−O)_3_ and Si(−O)_4_, are generated. It means that normal oxygenated Si atoms (Si(−O)_2_), also known as Si−(CH_3_)_2_, can be used to represent Si atoms on the main chain. As a result, the reduction of the peak area of Si−(CH_3_)_2_ instead of Si−O−Si is selected to quantitatively characterize the degree of degradation during multi-factor aging.

As can be seen in Figure 7a, with the addition of corona discharge and humidity, there is an obvious decrease in the absorption peak area of Si−(CH_3_)_2_. Somewhat different from the crosslinking density results, samples of group CH-35~85 °C possess the lowest peak area of Si−(CH_3_)_2_. It is probably due to the fact that the enhanced movement of molecules caused by heat promotes the cracking of the bonds on the main chain. Consequently, samples from groups (CH-35~85 °C) with a high average temperature tend to possess a high degradation degree.

#### 3.2.3. X-ray Photoelectron Spectra

**Figure 8:**
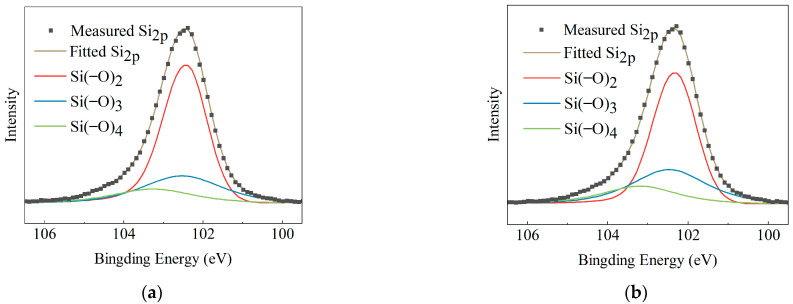

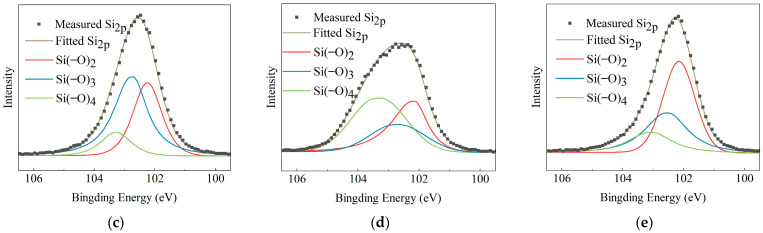
Fitting results of Si 2p spectra of HTV-SR samples under different aging conditions: (**a**) virgin; (**b**) N-35~85 °C; (**c**) CH-15~65 °C; (**d**) CH-15~85 °C; (**e**) CH-35~85 °C.

XPS analysis is carried out to analyze the changes in the basic structural units of HTV-SR during the aging process. The bound states of Si in the material are mainly composed of Si(−O)_2_, Si(−O)_3_, and Si(−O)_4_, corresponding to the binding energies of 102.1 eV, 102.8 eV, and 103.4 eV, respectively [16]. Then, a split-peak fitting operation is performed on Si 2p, and the content of each bound state is calculated. The results are shown in Figure 8 and Table 2.

It is worth noting that the content of Si atoms in the highly oxidized state (Si(−O)_3_ and Si(−O)_4_) is related to the degree of crosslinking reaction in the material [29]. According to Table 2, under different aging conditions, the crosslinking points of samples increase to different degrees. With the same temperature condition of 35~85 °C, the addition of corona discharge and humidity enables the content of highly oxygenated Si atoms to increase by 26.93%. It implies that corona oxidation makes it easier for HTV-SR to form crosslinking networks. Besides, samples (CH-15~85 °C) under temperature-cycling conditions with large ΔT have the maximum content of highly oxygenated Si atoms (60.4%), which is in accordance with crosslinking density results. It is further confirmed that the effect of temperature cycling promotes the aging of HTV-SR. Another interesting phenomenon is that the percentage of Si(−O)_4_ reaches 40% in CH-15~85 °C specimens (as shown in Figure 8d). It is probably due to the fact that the Si atom is crosslinked twice because of the aggressive oxidation allowed by surface defects, forming a number of SiO_2_ structures similar to those in silicone resins.

#### 3.2.4. Hardness and Elongation at Break

**Figure 9:**
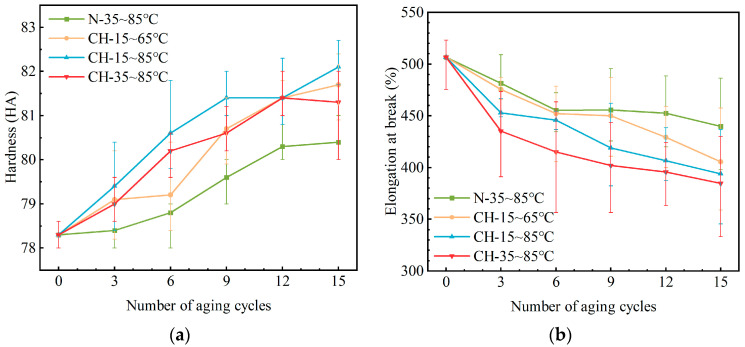
Mechanical properties of HTV-SR samples under different aging conditions: (**a**) hardness; (**b**) elongation at break.

The aging of HTV-SR at changing temperatures also has an effect on its mechanical properties [30]. Hardness and elongation at break are considered in this section. The measured data for these mechanical properties is shown in Figure 9. With the introduction of the corona and humidity, there is a significant increase in hardness and decrease in elongation at break, with a maximum change of 5.3% and 24.1%, respectively. Combined with the results of chemical properties, it implies that chemical structure and mechanical properties are closely related.

### 3.3. Electrical Properties

#### 3.3.1. Dielectric Spectra

**Figure 10:**
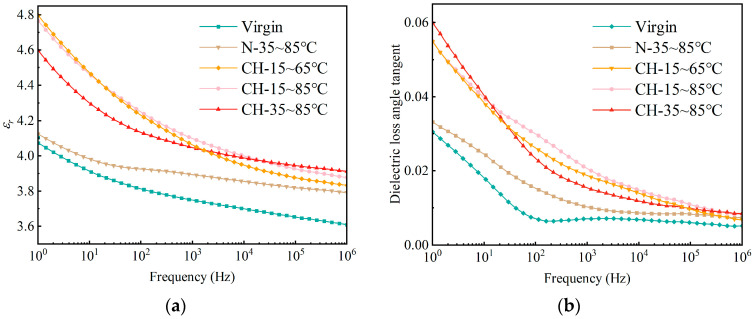
Real part of complex permittivity (**a**) and dielectric loss angle tangent (**b**) of HTV SR under different aging conditions.

The dielectric spectra of HTV-SR under different conditions are also investigated to evaluate its aging status. As shown in Figure 10a, it indicates that, after multi-factor aging, the real part of complex permittivity (*ε_r_*) increases significantly almost along the entire frequency range. The data of *ε_r_* in the low and medium frequency bands (10^0^ Hz to 10^4^ Hz), interestingly, vary depending on the temperature-cycling conditions. It is found that ε_r_ is lower for HTV-SR samples (CH-15~85 °C) with a larger ΔT. In the high frequency band (10^4^ Hz to 10^6^ Hz), the curve also increases slightly compared to the initial samples. Figure 10b shows that the dielectric loss (tan *δ*) in the low and medium frequency bands is strongly affected by corona discharge and temperature-cycling conditions, while the data in the high frequency band tends to be constant.

To further study the principle of the changes in dielectric spectra, the Havriliak-Negami (HN) dielectric relaxation model is adopted, and different relaxation processes are separated for the quantitative analysis [31]. The HN model with three relaxation processes is expressed in Equation (3).

(3) $$\varepsilon *=\varepsilon_{\infty }+\sum_{i=1}^{3}\frac{\Delta \varepsilon_{i}}{{\left[1+{\left(iw\tau_{i}\right)}^{m_{i}}\right]}^{n_{i}}}+\frac{\sigma_{DC}}{i\varepsilon_{0}w}$$

where ε* is the complex permittivity and *ε*∞ is the optical permittivity; Δ*ε*_i_ and *τ*_i_ are the strength and time constant of three relaxation processes, respectively; *m_i_* and *n_i_* are two indexes representing the dispersion degree of the relaxation process; and *σ_DC_* and *w* are the DC conductivity and the angular frequency, respectively [32].

**Figure 11:**
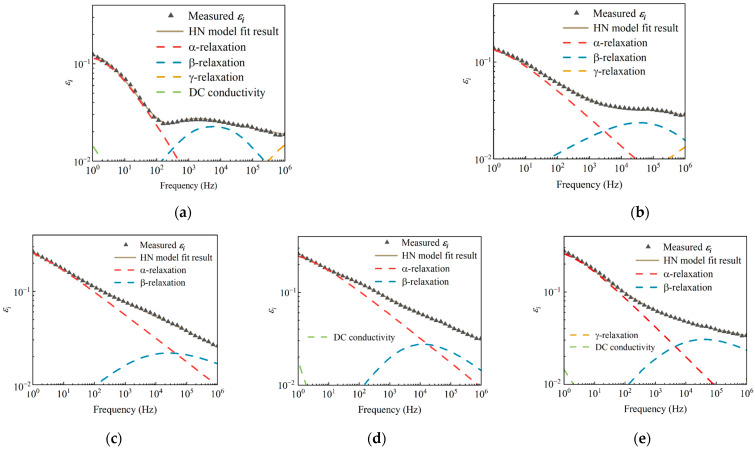
Fitting results of the HN model for the *ε_i_* curves of samples under different aging conditions: (**a**) virgin; (**b**) N-35~85 °C; (**c**) CH-15~65 °C; (**d**) CH-15~85 °C; (**e**) CH-35~85 °C.

Taking the spectra of the imaginary part of complex permittivity (*ε_i_*) of HTV-SR samples under different conditions as examples. As shown in Figure 11, the HN model provides a good fit to each dielectric spectrum curve. Moreover, the frequencies of peaks (10^−1^ Hz ≤ *f_p_* ≤ 10^6^ Hz) and the corresponding relaxation strengths (Δ*ε* ≥ 0.01) of three relaxation processes can be accessed by the HN model. Since the fitting results show that *γ*-relaxation, whose *f_p_* is much larger than 10^6^ and Δ*ε* is small, is not a focused relaxation process, then only *f_p_* and Δ*ε* of the two main relaxation processes are listed in Table 3.

Results show that the HTV-SR dielectric spectrum is composed of Maxwell–Wagner–Sillars (MWS) relaxation, *β*-relaxation, and *γ*-relaxation. With the addition of corona discharge and humidity, MWS relaxation and *β*-relaxation are improved when compared to virgin HTV-SR samples. Especially, it can be seen that MWS relaxation is enhanced with the increase in ΔT. Since different physicochemical properties between the inorganic filler and the polymer matrix might lead to interfacial incompatibility [33]. Then, the dynamics of charge transport in HTV-SR material can be affected. In this case, the rise of MWS relaxation is likely due to the change in interfacial polarization caused by various defects, as exemplified by holes, particles, and cracks shown by the results of surface morphology [34].

Regarding *β*-relaxation, there is a positive correlation between average temperature and the intensity of *β*-relaxation. It suggests that heat might facilitate dipole polarization, during which the molecular main chains of HTV-SR decompose to produce more low molecular weight (LMW) substances [13]. In addition, the variation of *β*-relaxation is in line with the FTIR results, again illustrating the role of accumulated heat in promoting the breakage of molecular chains. Additionally, *γ*-relaxation occurs at high frequency, which is mainly composed of electron polarization or chain segment vibration of HTV-SR under the influence of an electric field [35].

#### 3.3.2. Trap Properties

**Figure 12:**
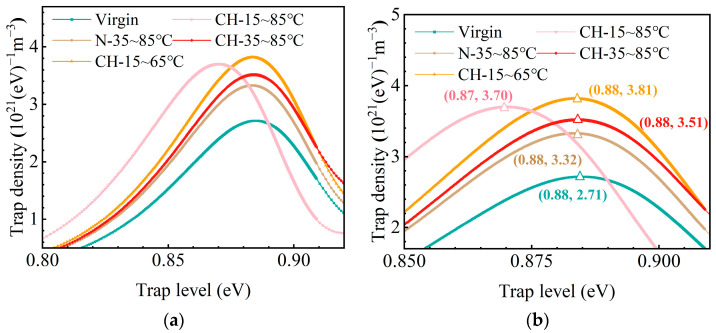
Trap properties (**a**) and specific trap density and energy level of the trap peak around 0.88 eV (**b**) of HTV-SR under different aging conditions.

To identify surface electron trap states of HTV-SR, the ISPD method is applied to measure the electron trap energy distribution. Figure 12 shows trap densities and trap levels of samples under different conditions. With the ISPD data, the electron trap density *N*(*E_t_*) can be calculated by the following equation:

(4) $$N(E_{t})=\frac{\varepsilon_{0}\varepsilon_{r}t}{kTf_{0}(E_{t})\delta Lq}\frac{dVs}{dt},$$

where *E_t_* is the depth of the electron trap, *f*_0_ (*E_t_*) is the rate of initial occupancy of traps; *V_s_* is the surface potential; *T* is the absolute temperature; *q* is the coulomb’s quantity of electron; *L* is the thickness of samples; *δ* is the thickness of the surface charge layer; and *ε*_0_ and *ε_r_* are the vacuum permittivity and the relative permittivity of samples, respectively [26].

As shown in Figure 12a,b, compared to virgin samples, there are different degrees of lifting in the trap density of samples after aging. Among them, the trap density of samples (CH-15~65 °C and CH-15~85 °C) increases significantly under temperature-cycling conditions with a below-average temperature. This is probably due to the physical and chemical defects on the surface formed by surface defects and the breakage of molecular bonds. In particular, when ΔT increases to 70 °C, there is an obvious reduction in the trap energy level corresponding to the peak of trap density, from 0.88 eV to 0.87 eV. Due to the fact that physical defects mostly correspond to shallow traps while chemical defects correspond to deep traps [36]. Such cavity defects on the surface of the material could possibly cause a change in the way charge dissipation occurs, from deep to shallow energy levels of trap properties.

## 4. Discussion

As an external insulating material for composite insulators, it is essential to conduct an accelerated deterioration analysis under multi-factor aging, considering temperature cycling. Firstly, a crosslink-decomposition model is proposed to study the effects of corona discharge, humidity, and temperature cycling on the aging mechanism. Then, aging behaviors with different ΔT are investigated and summarized. Finally, the effectiveness of artificial aging is demonstrated.

### 4.1. Multi-Factor Aging Mechanism of HTV-SR

**Figure 13:**
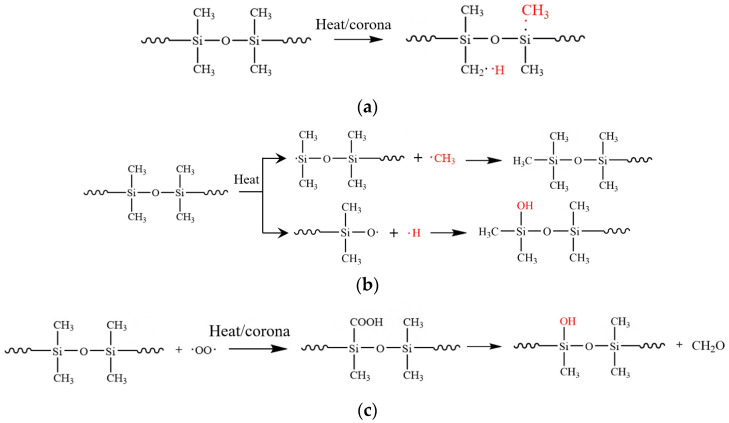

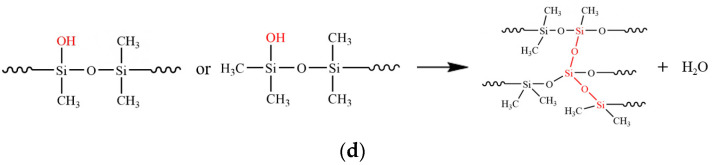
Reactions of HTV-SR during the aging process: (**a**) generation of free radicals; (**b**) decomposition reaction of the main chain (−OH at the end of the chain); (**c**) direct oxidation of the side chain (−OH at the middle of the chain); (**d**) crosslinking reaction of the side chain (−OH at different locations).

The multi-factor aging test in this study is in an alternating temperature, high moisture, and corona discharge environment, so a chemical crosslink-degradation model is proposed to achieve an accessible mechanism analysis. Two main reactions, such as crosslinking and degradation, and some auxiliary reactions, such as the generation of free radicals [15] and the direct oxidation of side chains, are shown in Figure 13a–d. As shown in Figure 13a, to begin with, polydimethylsiloxane (PDMS) generates free radicals under corona and thermal stress. Then, under high-temperature conditions, the main chain is cleaved and then combined with free radicals to produce silanol and LMW substances, as shown in Figure 13b. Meanwhile, as shown in Figure 13c, under the effects of corona and thermal oxidation, the side chains of PDMS undergo redox reactions to produce silanol directly. With −OH bands in different positions, silanol groups will crosslink under the dehydration process, producing Si atoms at different oxidation states in Figure 13d, which is verified by the XPS test results.

Comparing the results of crosslinking density, FTIR results, and XPS tests, it is demonstrated that the crosslinking reaction in Figure 13d is the main reaction of HTV-SR in the early aging period under different conditions. It can be attributed to the generation of reactants such as silanol under the conditions of corona and heat. When the crosslinking density grows rapidly to a high level, more Si−O−Si bonds are available for degradation. Afterwards, the degradation reaction in Figure 13b dominates in the later aging period of HTV-SR. The analysis implies that crosslinking and degradation are synergistic, but the proportion of the two is different with the aging time.

**Figure 14:**
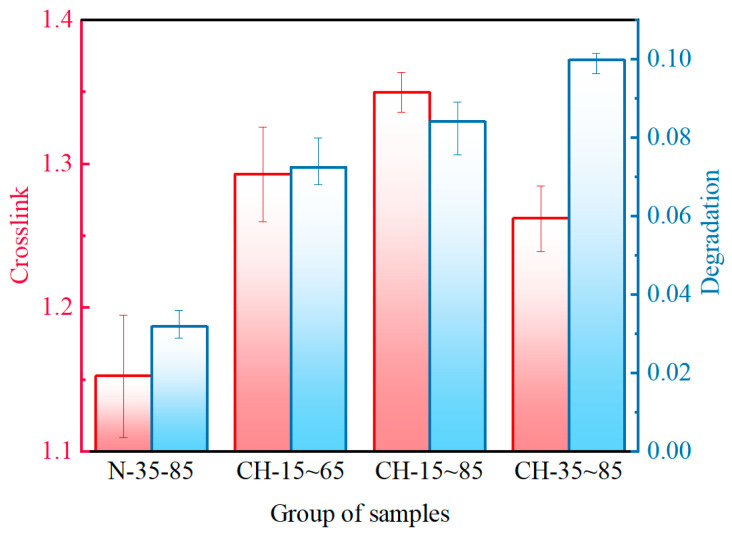
Crosslink-degradation degree of samples after 15 cycles of aging.

To determine how corona discharge, humidity, and temperature cycling affect the aging mechanism of HTV-SR, crosslink-degradation degrees of the “N” and “CH” groups are considered for comparison. As can be seen in Figure 14, both crosslinking and degradation degree of samples in “CH” groups are higher than those in the “N” group. It is due to the fact that corona and moisture exposure enable molecular chains to be oxidatively crosslinked and Si−O−Si structures to be produced, thus promoting the degradation reaction (Figure 13b). Furthermore, by comparing crosslink-degradation degrees of samples between different “CH” groups, the effects of temperature cycling on the aging mechanism of HTV-SR are discussed. When ΔT is 50 °C, samples (CH-35~85) with a higher average temperature possess a higher degradation degree and a lower crosslinking degree. It is due to the fact that degradation reactions mainly occur at high temperatures, which is consistent with *β*-relaxation in dielectric spectra. As ΔT increases to 70 °C, the crosslinking degree clearly increases, while the degradation degree does not. This is because in-depth corona oxidation in Figure 13c and the crosslinking reaction allowed by a large ΔT promote the crosslinking while a low average temperature prevents the degradation, facilitating the formation of crosslinking networks. In this case, a high degree of crosslinking occurs, where the relative content of highly oxygenated Si atoms, especially Si(−O)_4_, reaches 40%.

### 4.2. Effects of Temperature Cycling on Aging Behaviors of HTV-SR

**Figure 15:**
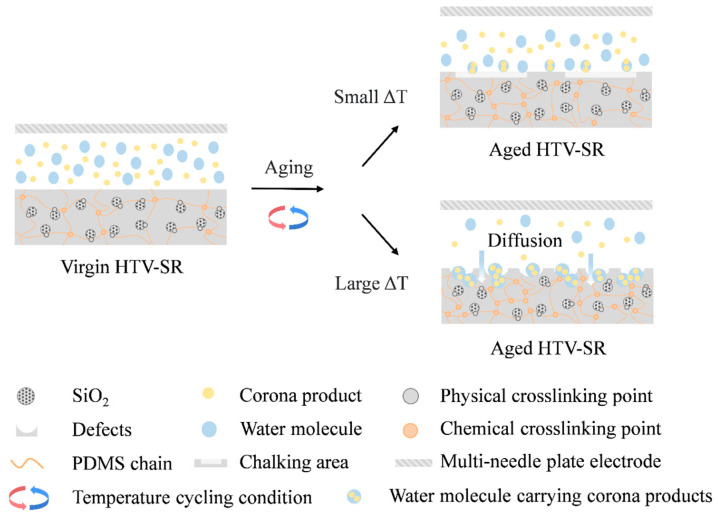
Aging behaviors of HTV-SR under different ΔT.

Different crosslink-degradation degrees of HTV-SR contribute to its diverse internal structure, which has a great influence on its aging behaviors. Then, the aging behaviors of HTV-SR under different ΔT are summarized in Figure 15.

When ΔT is small (50 °C), aged samples tend to have a high degradation degree and a low crosslinking degree. This indicates that plenty of PDMS chains crack and produce silanol, LMW, and other small molecules, making it easier to form the pulverized layer. Then, some chalking corona rings caused by corona discharge emerge on the sample surface. In particular, the aging behaviors of samples with a below-average temperature (15 °C) are more severe than those with only high temperatures, which emphasizes the necessity of low temperatures in temperature cycling.

As ΔT increases to 70 °C, the aged samples possess a lower degradation degree and a higher crosslinking degree. Then, the intermolecular links become closer, and a crosslinking network is formed, making HTV-SR harder but easier to crack under temperature cycling conditions. Therefore, various defects, such as holes, cracks, particles, etc., emerge on the sample surface. Besides, the aging phenomena are more pronounced with the promotion of corona discharge and humidity. This is probably due to the severe water condensation under large ΔT and high absolute humidity conditions [23], which amplifies the accelerating effect of moisture on aging. With a large amount of condensed water molecules and serious surface defects, HTV-SR possesses the performance of “water locking.” Then, corona products, such as O_3_ and HNO_3_ [9], are adsorbed by the liquid water on the surface of the material, causing corona aging to no longer stay on the surface but further invade the interior. Moreover, the HTV-SR material is usually reinforced by adding silica materials, during which a physical crosslinking network is formed by hydrogen bonds between the main chain and SiO_2_ [10]. Despite the unchanged physical crosslinking points, chemical crosslinking points increase because of the oxidizing effect within the material, enhancing the apparent crosslinking density. The process of internal aging further contributes to the deterioration of HTV-SR. As a result, due to severe surface defects, hydrophobicity declines. Additionally, the presence of surface defects brings about unique electrical properties such as high interface polarization and shallow trap energy levels.

### 4.3. Comparison between Artificial Aging and Natural Aging

On-site experience shows that under long-term exposure to various stresses, the HTV-SR material used for the sheath and skirt tends to fail before the core rod does. Then, some obvious aging behaviors, such as chalking and cracking, emerge on the material surface, which help guide site maintenance. The structural changes in this aging experiment are highly consistent with those observed in the aging of insulators in the field [37]. Besides, as discussed in mechanical properties, the elongation at break of HTV-SR eventually decreased by 24.1%. In the existing literature studying the performance of naturally aged composite insulators [38], the tensile strength of insulators aged in the field for 7 years decreased by approximately 25%, from 5.5 MPa to 4.1 MPa. By approximate comparison, the change in mechanical properties of HTV-SR illustrates the validity of the multi-factor artificial aging method, considering temperature cycling. The results also provide evidence that corona products and condensed water molecules under temperature-cycling conditions play an accelerating role in the internal aging of materials.

## 5. Conclusions

In this study, for regions with a large temperature difference (ΔT) between day and night, a multi-factor accelerated aging platform considering temperature cycling, corona discharge, and humidity is conducted on HTV-SR. The aging behavior considering surface morphology, hydrophobicity, chemical, mechanical, and electrical properties is experimentally explored, and some details are as follows:

(1) A multi-factor aging technique for insulator HTV-SR is proposed, which takes the alternating thermal stress into account and is more in line with the on-site aging process of HTV-SR. Compared to existing aging methods, the method in this paper investigates the influence of ΔT and the related water condensation phenomena.
(2) The aging degree of HTV-SR is largely affected by ΔT. Under small ΔT (50 °C), chalking corona rings emerge on the sample surface. Under large ΔT (70 °C), severe surface defects, poor hydrophobicity, high interfacial polarization, and shallow trap energy levels are obtained. It is due to the intrusion of water molecules and corona products into the inside of HTV-SR material, further accelerating the aging.
(3) It is concluded that crosslinking and degradation chemical reactions are synergistic but vary with aging. When ΔT increases, the crosslinking degree rises while the degradation degree declines. The crosslink-degradation model with different occupancy ratios plays a decisive role in the aging behavior of HTV-SR.

## Figures and Tables

**Table 1:** Multi-factor aging test program considering temperature cycling.

Group	T_range_ (°C)	T_mean_ (°C)	ΔT (°C)	Corona (C)
				Humidity (H)
N-35~85 °C	35–85	60	50	×
CH-15~65 °C	15–65	40	50	√
CH-15~85 °C	15–85	50	70	√
CH-35~85 °C	35–85	60	50	√

**Table 2:** Relative compositions of Si−O structures of samples under different aging conditions.

Sample	Si(−O)_2_	Si(−O)_3_	Si(−O)_4_	Highly Oxidized Si (Si(−O)_3_ + Si(−O)_4_)
Virgin	80.00%	13.60%	6.40%	20.00%
N-35~85 °C	76.93%	15.38%	7.69%	23.07%
CH-15~65 °C	39.91%	44.84%	13.45%	58.29%
CH-15~85 °C	39.60%	20.40%	40.00%	60.40%
CH-35~85 °C	50.00%	33.00%	17.00%	50.00%

**Table 3:** Dielectric relaxation parameters of HTV-SR.

Group	MWS Relaxation		*β*-Relaxation	
	*f_p_* (Hz)	Δ*ε*	*f_p_* (Hz)	Δ*ε*
Virgin	0.131	0.819	6724	0.021
N-35~85 °C	0.519	0.960	43,262	0.022
CH-15~65 °C	0.218	1.735	34,572	0.022
CH-15~85 °C	0.255	1.825	16,939	0.027
CH-35~85 °C	0.562	1.687	58,715	0.031

## Data Availability

Data presented in this study are available on request from the corresponding author.
